# Relevant heating of the quiet solar corona by Alfvén waves: a result of adiabaticity breakdown

**DOI:** 10.1038/s41598-019-50820-x

**Published:** 2019-10-03

**Authors:** D. F. Escande, V. Gondret, F. Sattin

**Affiliations:** 1 Aix-Marseille Université, CNRS, PIIM, UMR 7345, Marseille, France; 2 École Normale Superieure, Physics Department, Paris, France; 3 Consorzio RFX (CNR, ENEA, INFN, Università di Padova, Acciaierie Venete SpA), Padova, Italy

**Keywords:** Solar physics, Solar physics

## Abstract

Ion heating by Alfvén waves has been considered for long as the mechanism explaining why the solar corona has a temperature several orders of magnitude higher than the photosphere. Unfortunately, as the measured wave frequencies are much smaller than the ion cyclotron frequency, particles were expected to behave adiabatically, impeding a direct wave-particle energy transfer to take place, except through decorrelating stochastic mechanisms related to broadband wave spectra. This paper proposes a new paradigm for this mechanism by showing it is actually much simpler, more general, and very efficient. Indeed, for measured wave amplitudes in the quiet corona, ion orbits are shown to cross quasi-periodically one or several slowly pulsating separatrices in phase space. Now, a separatrix is an orbit with an infinite period, thus much longer than the pulsation one. Therefore, each separatrix crossing cancels adiabatic invariance, and yields a very strong energy transfer from the wave, and thus particle heating. This occurs whatever be the wave spectrum, even a monochromatic one. The proposed mechanism is so efficient that it might lead to a self-organized picture of coronal heating: all Alfvén waves exceeding a threshold are immediately quenched and transfer their energy to the ions.

## Introduction

A long-standing problem is to explain why the solar corona has a temperature several orders of magnitude higher than the photosphere^1–5^. There is a number of effects, e.g. shocks, shock-shock collisions, cumulative effects, reconnections etc., that are observed and theoretically proved to be responsible for heating different parts of corona^1^. Among these, ion heating by Alfvén Waves (AWs) looks as an efficient mechanism^4,5^, since these waves propagate effectively in the corona^6–8^, and with a high energy flux. This paper deals with the quiet corona and coronal holes considered by McIntosh *et al*.^9^ in their paper “Alfvénic waves with sufficient energy to power the quiet solar corona and fast solar wind”, where they extended to the quiet corona and coronal holes a statement initially made for the chromosphere^10^. They show that the energy flux of AWs potentially accounts for the whole energy budget in the region of the quiet corona they consider. The fact that this energy flux is not larger than the whole energy budget suggests that the waves are fully absorbed.

Here we provide a strong backup to the mechanism of ion heating by AWs by introducing a new paradigm for AW-ion interaction: First, despite the extremely large separation of scales existing between the low-frequency coronal AWs and the ion cyclotron frequency^11,12^, ion motion in a spectrum of these waves is not adiabatic at all, because it involves, for the measured wave amplitudes, one or several slowly pulsating separatrices in phase space, which force particles to cross them in a quasi periodic way. Each separatrix crossing destroys adiabatic invariance, since the period is infinite there, yielding irreversible energy transfer from the wave, and thus particle heating, without invoking any stochastic decorrelation. This is a new aspect of neo-adiabatic theory (see the works^13–17^ and references therein), which is of general interest. Second, this heating occurs no matter how narrow the wave spectrum: a monochromatic spectrum is relevant, and even very efficient. This contradicts the widespread belief that only decorrelation induced by a broadband spectrum can provide an efficient direct heating of the ions by AWs^11,12,18–22^. Third, self-organization is likely at work here. Indeed, the heating mechanism is so efficient that it may quench AWs, which would explain why the energy flux in the quiet corona corresponds to the whole thermal energy budget, as argued in the work by McIntosh *et al*.^9^.

We start our analysis with this *new paradigm in Alfvénic heating* implied by the efficiency of a single wave. In the low-*β* solar environment (*β* ≤ 0.01)^2^, the interaction of ions with AWs can be described by a non-self-consistent test-particle approach, where ions are mutually independent particles interacting with a prescribed wave^11,12,18,23–25^. The ion-electron Coulomb collision frequency is ≈10^−2^ ÷ 10^−3^ Hz, overlapping the lower side of the range of AW frequencies. Therefore, ions may be considered as collisionless, which enables a Hamiltonian description (this might be questionable in dense regions of the corona). The Hamiltonian system is non-autonomous, with a temporal variation parametrized by the ratio between AWs frequency *ω* and the ion cyclotron one Ω:*ω*/Ω ≪ 1^11,12^. Indeed, under lower corona conditions, Ω lies in the kHz range, while most of the spectrum of AWs lies in the mHz range (possibly up to the Hz range^26^). This implies that the linear resonance condition between an AW and the cyclotron motion of magnetized ions–the most straightforward and effective mechanism for energy transfer–is not fulfilled there, and Doppler shift is not strong enough to fill the gap.

Consistently with the existing literature, we consider an equilibrium magnetic field directed along axis *z* with constant amplitude: ${{\bf{B}}}_{eq}={B}_{0}\hat{{\bf{z}}}$, and an AW linearly polarized along *y* propagating obliquely. In the frame of reference moving with the Alfvén speed *u*_*A*_, there is no electric field, and the magnetic perturbation is described by the vector potential: **A** = (*b*_*ω*_/*k*_*z*_sin*ψ*, 0, 0), *ψ* = *kx* + *k*_*z*_*z*, where *b*_*ω*_ is the amplitude of the magnetic perturbation. Finally, the angular frequency of the wave is *ω* = *u*_*A*_*k*_*z*_.

**Figure 1:**
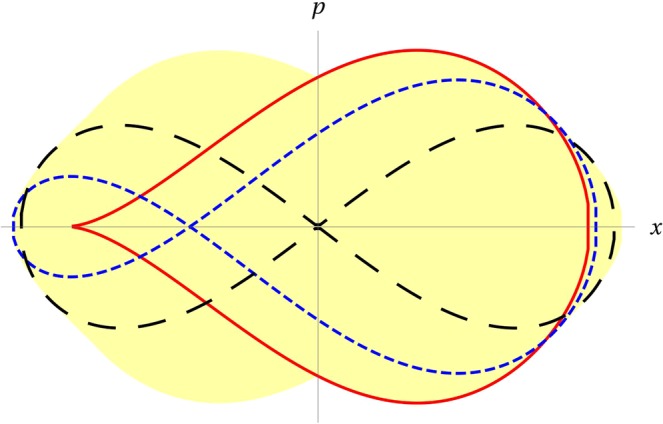
Snapshots of the slowly pulsating separatrix. The three curves represent the separatrix at different times. Red solid curve, the separatrix is just appearing with a single right lobe; blue short-dashed curve, a left lobe is developing; black long-dashed curve, the two lobes are symmetrical with respect to the *p* axis; during the next half-period, the separatrix evolves with mirror symmetry. The yellow domain is the total area swept by the separatrix throughout one period. The figure has been produced with *A* = 3.

The wave amplitude, written in dimensionless form (see the file Supplementary Equations for a detailed derivation), is

1 $$A=(k\rho )({b}_{\omega }/{B}_{0})({u}_{A}/{c}_{s})$$

where *ρ* is the ion thermal Larmor radius and *c*_*s*_ the ion sound speed. Realistic values for these ratios are now discussed. Figure 1 of Chandran^27^ provides the values (~2 Mm/s) for the measured Alfvén speed between 1.05 and ~1.35 solar radii. Also the middle of the third column of page 1194 of Tomczyk *et al*.^28^. The latter reference provides the estimate (~0.2 Mm/s) for the sound speed, which corresponds to a proton with a temperature of about 1 MK. These estimates of both speeds are confirmed between equations (13) and (14) of Muravski and Musielak^29^, and by Fig. 1b of Esser *et al*.^30^. This yields (*u*_*A*_/*c*_*s*_) ≈ 10. Figure 2a of Esser *et al*.^30^ provides ${b}_{\omega }/{B}_{0}\,\gtrapprox \,0.1$. The estimates given at the end of section 3 of Chandran^27^ and in the middle of the second column of page 504 of Chandran *et al*.^12^ suggest $k\rho \,\lessapprox \,1$; indeed, the energy spectrum of AWs cascades in the perpendicular wavenumber space, evidencing a stronger damping for *kρ* ≥ 1, transitioning to kinetic AWs near this threshold^31^. Summing it all up, it sounds reasonable to take *A* ≈ 1 as the typical wave amplitude.

**Figure 2:**
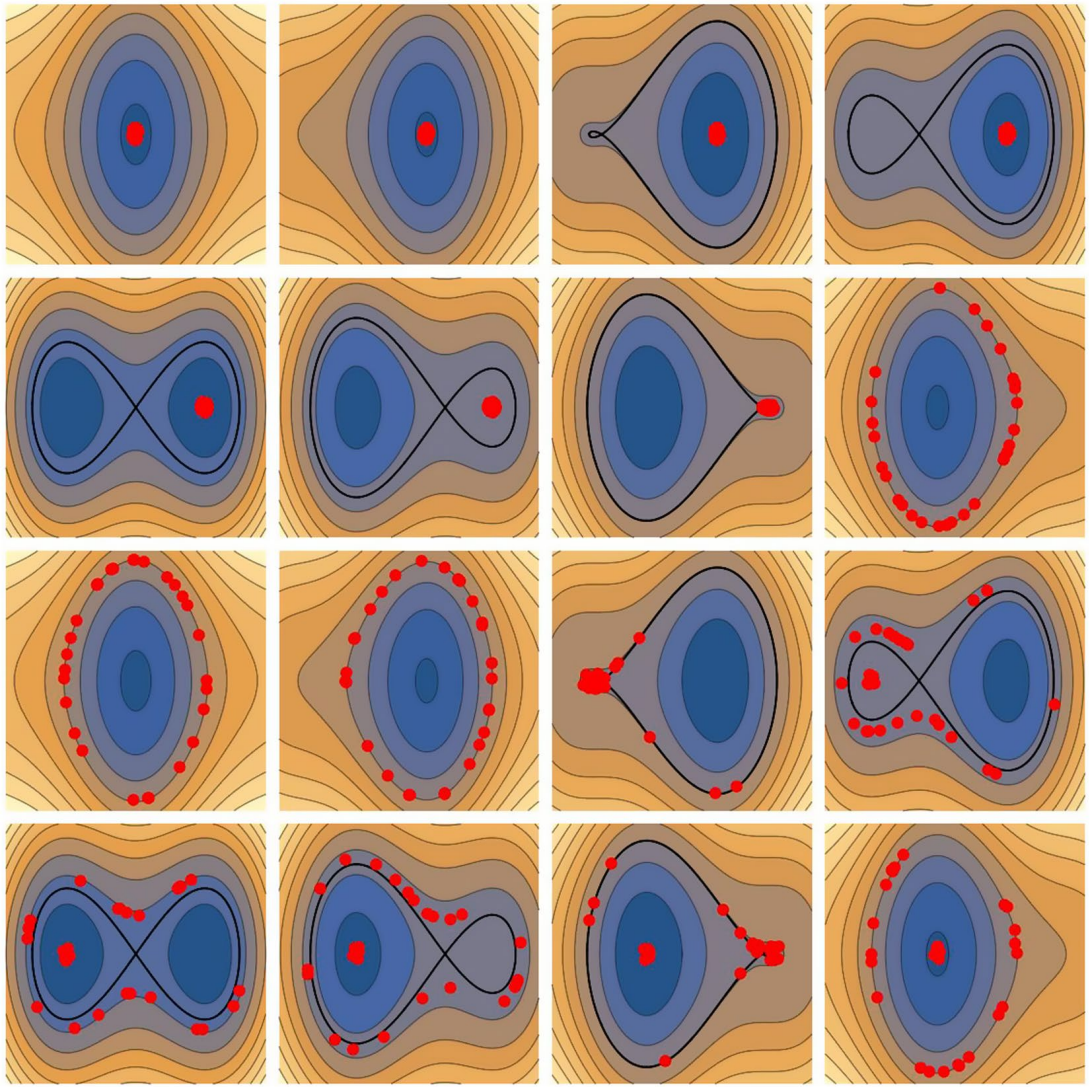
Separation in energy reached by abrupt separation of orbits. Frames show snapshots at increasing times (from left to right and from top to bottom) of the phase-space (*x*, *p*) position of a bunch of particles (red dots). The contour levels are those of *H*_1_(*t*) (Eq. 2) with *ϕ* = *π*/2. Particles are initialized close to the origin; they stay grouped together until the lobe disappears (7 *th* frame); then distribute along the iso-energy level. After the lobe and the separatrix reappear (11 *th* frame), particles are separated into two distinct groups, one with the original energy, and another with a higher energy.

Let us consider a cold ion, initially at rest in the reference frame of the sun. Its dynamics in the former reference frame has been studied by Chen, Lin and White^23,24^, which already found a heating process for *ω*/Ω small, but still much larger than its coronal value. These references show that the effective ion dynamics is one-dimensional along the x-axis, and generated by the dimensionless Hamiltonian *H*_1_: (see the file Supplementary Equations)

2 $$\ddot{x}=-\,x+A\,\cos (x-\omega t+\varphi ),\,{H}_{1}=\frac{{p}^{2}}{2}+\frac{{x}^{2}}{2}-A\,\sin (x-\omega t+\varphi )=\frac{{p}^{2}}{2}+V.$$

The potential term *V* in Eq. 2 is that of a harmonic oscillator modulated by a sinusoidal term (where *ϕ* is an arbitrary phase). At any fixed time, for any value of *A*, *dV*/*dx* admits at least one root, corresponding to a minimum of *V*. Several roots exist whenever *A* > 1, during a fraction of the wave period *T* = *ν*^−1^ = 2*π*/*ω*, and then maxima and minima alternate. In phase space, a maximum of *V* corresponds to an X-point, starting point of a separatrix (see Fig. S1 in the file Supplementary Figure). Figure 1 yields a pictorial view of the phenomenology involved while a single separatrix is evolving. As the right lobe of the separatrix decreases, the area it loses goes partly in the left lobe and partly outside the separatrix. This brings an abrupt separation of orbits–depicted in Fig. 2. Numerical inspection shows that it is necessary to go up to *A* ≈ 5 in order for the second maximum to appear. We only consider the case with a single maximum (*A* < 5): from the one hand, accounting for several separatrices does not add qualitatively any new physics, and secondly already a single separatrix crossing will prove to bring so fast a transfer of the wave energy to the ions, that AWs might be quenched before reaching higher amplitudes. This further substantiates our estimate: *A* ≈ *O*(1), and shows that self-organization is at work.

Paper^25^ shows that the acceleration of an ion does not occur permanently, but through a sequence of superadiabatic accelerations at the occurrence of a special phase matching between the wave and the ion motion, and ions look as demagnetized during their acceleration. This is a mere consequence of separatrix crossing. Indeed, during this process the orbit takes on a large “Larmor radius” (the excursion of *x* is large, see Fig. 2). Since it comes close to the X-point, it feels the exponential divergence related to it, a fact reference^25^ exhibits analytically and numerically. Furthermore, when the orbit is close to the X-point, the corresponding phase of the wave corresponds to the most negative value of *d*^2^*V*/*dx*^2^, another fact exhibited in this work.

Numerical calculations of orbits were performed in the works^11,12,18,23–25^. We now use them to exhibit the above abrupt separation of orbits and its consequences. The numerical integration of Hamilton’s equations is performed using the 6*th*–order symplectic partitioned Runge-Kutta algorithm built into Mathematica software.

**Figure 3:**
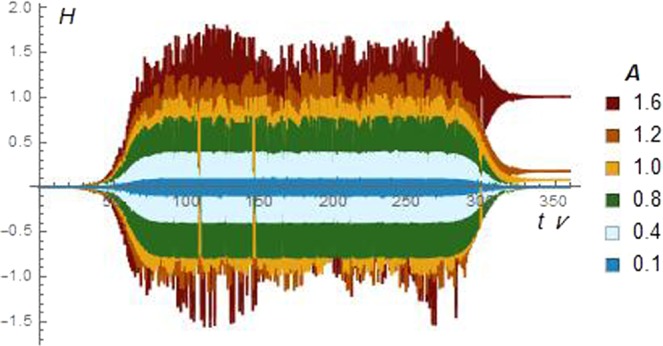
Particle energy *H*_1_ vs. time. Several values of the amplitude *A* are considered. Only in the cases *A* ≥ 1 there is a net gain in the final particle energy. These results have been obtained at a fixed phase of the wave; by varying the phase, the final value of *H*_1_ may change at most by 30%.

Equation 2 shows that a particle initially at rest gains some kinetic energy for any finite value of *A*. This is the ion pick-up mechanism by the wave, well known in literature^32,33^. By itself, this is not a proper heating since the energy flow may be reversed and returned back fully to the wave once the interaction is switched off. In order to make it irreversible, it must be supplemented by collisions or by the chaos due to the pulsating separatrix (see the works^16,17^ and references therein). Figure 3 makes visual the net effect due to this latter mechanism. In this case, the waveform has been modulated by a shape function *f*:*A* → *A* × *f*(*t*),

3 $$f(t)=\frac{1}{4}[1+\,\tanh (\frac{t-{t}_{s}}{\Delta })][1+\,\tanh (\frac{{t}_{f}-t}{\Delta })],$$

such that there is no wave for *t* ≪ *t*_*s*_ and *t* ≫ *t*_*f*_. We were careful in switching the modulation *f* on and off extremely slowly, so that the electric field contribution could still be neglected in the equations of motion. The asymptotic value of the energy *H*_1_(*t*) from Eq. 2 shows whether the energy transfer to the ions was irreversible. Figure 3 plots *H*_1_(*t*) for several choices of *A*. There is a net gain in the particle kinetic energy only for those cases where *A* > 1.

**Figure 4:**
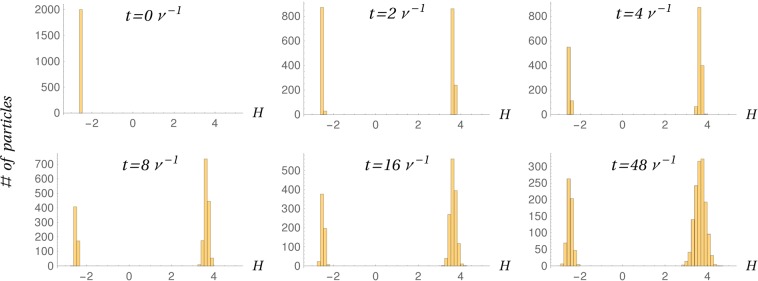
Histogram of the energy distribution at different times. We bin the energy of a set of 2000 particles initially almost at rest (*x*, *p* ≈ 0), with *A* = 3, *ω* = 0.003. All the simulations were run for a fixed phase of the wave such that particles are initially close to the O-point. Another example, for a different value of the phase, is reported in the Fig. S2 in the file Supplementary Figures.

The separatrix sweeps the whole yellow area of Fig. 1 within one wave period, which is therefore the characteristic time scale for the decorrelation of trajectories, by analogy with the simple case considered by Bruhwiler and Cary^13^. Figures 4 and S2 in the file Supplementary Figures, show that the abrupt separation of orbits leads to the sudden formation of a high-energy tail in the distribution of energies. As soon as this tail is created, it starts to diffuse, as well as its low-energy counterpart, and the two distributions are expected to merge completely after a time ~*ω*^−3^ (see Bruhwiler and Cary^13^ and Eq. 5.7.22 in Lieberman and Lichtenberg^16^). In view of the possible quenching, this may be too long a time for small *ω*’s, but–once energized–ions may reach faster a thermal distribution due to other decorrelating mechanisms, such as collisions. Running the simulation with *A* sensibly smaller than 1 does not produce any appreciable spreading of the initial energy distribution, since there is no abrupt separation of trajectories any longer.

Stroboscopic plots enable viewing the macroscopic consequence of the abrupt separation of orbits in phase space. One instance has already been produced in Fig. 2. Figure S3 in the file Supplementary Figures and Supplementary Movie SMV1, contain another example where two orbits initially close to each other and to the separatrix then depart: one of them lands into the left lobe and the other one outside of the separatrix. By integrating over longer times we would see that a single orbit does fill up the domain swept by the pulsating separatrix (Fig. S4 in the file Supplementary Figures). By contrast, two particles with the same initial conditions stay together for *A* < 1 (Fig. S5 in the file Supplementary Figures).

The above results show that an initially cold particle, *when A is larger but of order unity, after interacting with a single AW for as short a duration as a double wave cycle, may gain a finite amount of energy E*_*f*_ ≈ *O*(*A*)~*O*(1). According to Fig. 3, *ions reach energies of the order of the coronal temperature* (in dimensioned units *E*_*f*_ = *k*_*B*_*T*, where *k*_*B*_ is the Boltzmann constant, and *T* is the measured coronal ion temperature).

We now show that AWs are likely to be quenched in the ion acceleration process. In two wave periods a part of the Poynting energy flux brought by the wave is absorbed by the ions on a length equal to two wavelengths *λ*. The ratio of the thermal flux injected into ions to the electromagnetic flux is then 2*λnk*_*B*_*T*/[4*πb*_*ω*_^2^*u*_*A*_/*ωμ*_0_] = *β*(*B*_0_/*b*_*ω*_)^2^ ≈ *O*(1) in the regions where measurements are available. Therefore, AWs are almost completely absorbed in the process if the ratio is close to 1. While our model assumes their amplitude is constant, in reality they are likely to be quenched at the end of acceleration process of the ions, because of conservation of energy. We thus expect the following self-organization: only AWs with *A* lesser or marginally larger than unity could be observed. Rephrased in a more intuitive way, forgetting about other possible heating mechanisms, the temperature of the corona adapts exactly to the amplitude of the AWs: AWs are continuously produced and quenched by transferring their whole energy to ions. This permanent quenching would explain why the energy flux in the quiet corona corresponds to the whole energy budget, and not more, in the work by McIntosh *et al*.^9^.

Let us now turn to the case of a broad spectrum, which was already proved to provide an efficient heating in works^11,12,18–20^. Neo-adiabatic theory provides a simple interpretation of this heating, where the assumption of stochastic phases of the components of the spectrum^11,12,19,20^ becomes optional. Indeed, again the extremely large separation of scales existing between the low-frequency coronal AWs and the ion cyclotron frequency^11,12^ justifies the consideration of the frozen potential of the waves. Typically, such a potential has several maxima, and each of them comes with a frozen separatrix (See Supplementary Movie SMV2). Unfreezing time, these separatrices slowly appear and disappear, forcing again particles to cross them, which destroys adiabatic invariance, and yields irreversible energy transfer from the wave, and thus produce high energy particles over a few quasi-periods (see Fig. S6 in the file Supplementary Figures). These particles then thermalize, for instance through chaos or through collisions, which may turn out to be more efficient. The degree of heating does not depend critically from the shape of the spectrum (there is roughly a threefold increase in the energy gain when passing from monochromatic to broad spectra–Fig. S7 in the file Supplementary Figures).

We now discuss the regions where measurements are not available. We stress that our theory has a broad applicability, which may make it relevant in other parts of the corona. It only needs the amplitude of AWs to cross a threshold for the existence of an efficient transfer of energy from the waves to the particles: two wave periods typically. This decreases the weight of peculiarities of wave propagation, boundary conditions, etc… The title “Chromospheric Alfvénic waves strong enough to power the solar wind” of De Pontieu *et al*.^10^ extends the claim of McIntosh *et al*.^9^ done for the corona. This suggests our mechanism is working also there. If the production of AWs is strong enough to reach the threshold of application of our theory, the quenching mechanism may be at work too, which would bound the amplitude of the waves. The competition between drive and quenching would lead to a limit cycle if a single AW is present. Since a single wave may be described as a harmonic oscillator, one reaches a dynamics very similar to that described by the Van der Pol equation in chap. 16 of Ryutova^1^.

In summary, the present study shows that an initially cold ion, after interacting with one or more AWs may gain a finite amount of energy *E*_*f*_ which is of the order of the coronal temperature: *E*_*f*_ ≈ *O*(*A*) ≈ *O*(1) (in dimensioned units *E*_*f*_ = *k*_*B*_*T*, where *k*_*B*_ is the Boltzmann constant, and *T* is the measured coronal ion temperature). It is true heating, since the energy flow is irreversible, and takes place almost immediately, within two wave cycles, hence time scales should not be an issue. Requirements upon length scales, too, do not appear stringent: the only requisite is that length scales involved (including AW wavelength) be order of or larger than the hot ion Larmor radius. The proposed mechanism does not require any resonance condition, is active for very low wave frequencies, and for monochromatic spectra (since it does not need random wave phases as decorrelation mechanisms). The only mandatory requirement is a minimum amplitude of AWs, which is found to be of the same order of magnitude as those observed. Actually, since energy is very effectively drained from large-amplitude AWs, these may be strongly quenched, and the assumption of fixed-amplitude might be no longer correct at the end of the heating process. Although precise details are affected by the shape of their spectrum, we thus expect that only AWs with amplitude *A* lesser or marginally larger than unity could be observed.

A preliminary version of this work was made available at the repository https://hal.archives-ouvertes.fr/hal-01929112.

## Supplementary information

Video SMV1

Video SMV2

Supplementary Equations

Dataset1

## Data Availability

Mathematica source codes used in the simulations are available from authors upon request.
