# Supplementary material for: Relevant heating of the quiet solar corona by Alfvén waves: a result of adiabaticity breakdown
Source: Sci Rep. 2019 Oct 3;9:14274. doi: 10.1038/s41598-019-50820-x (PMC6776755; doi:10.1038/s41598-019-50820-x)
Supplement: Supplementary file 3 — Supplementary Equations [file 41598_2019_50820_MOESM3_ESM.pdf]

Relevant heating of the quiet solar corona by Alfvén waves : a result of adiabaticity breakdown

D.F. Escande,[1] V. Gondret,[2] and F. Sattin[3]
[1] Aix-Marseille Université, CNRS, PIIM, UMR 7345, Marseille (France)
[2] École Normale Superieure, Physics Department, Paris (France)
[3] Consorzio RFX (CNR, ENEA, INFN, Università di Padova, Acciaierie Venete SpA), Padova (Italy)

Supplementary equations

Supplementary Equations

The reader may refer to [L. Chen, Z. Lin and R. White, Phys. Plasmas 8, 4713 (2001)] for the original calculations.

The full 3-dimensional Hamiltonian of a magnetized charged particle in the wave' reference frame writes

$$H = \frac{1}{2m}\left(p_x - q\frac{b_\omega}{k_z}\sin\psi\right)^2 + \frac{1}{2m}\left(p_y - qB_0 x\right)^2 + \frac{1}{2m}p_z^2 \ , \qquad \psi = k\,x + k_z\,z + \psi_0 \tag{S1}$$

As long as we consider particle velocities much smaller (in the laboratory frame) than the Alfvén velocity, we may set

$$p_z \approx -u_A m\,, \quad z \approx -u_A\,t \tag{S2}$$

Furthermore, p_y is a constant of motion and may be set to zero by an appropriate choice of the reference frame.

When $b_\omega = 0$ the ion dynamics reduces to Larmor rotation (plus translation along z): the total excursion along x is twice the Larmor radius.

Notice that during motion, some energy is ultimately transferred to the parallel direction, because of the coupling between the degrees of freedom in (S1) and of Coulomb collisions (unaccounted for in Eq. S1 but nonetheless present). However, measurements as well as numerical studies show that the energy flow into the perpendicular direction overwhelms that into the parallel one, which justifies approximation (S2) and the neglect of collisions.

With assumptions (S2)—and discarding the arbitrary phase--Eq. (S1) writes

$$H = \frac{1}{2m}\left(p_x - q\frac{b_\omega}{k_z}\sin(k\,x - \omega\,t)\right)^2 + \frac{q^2}{2m}B_0^2 x^2 + \frac{1}{2}m u_A^2 \ , \quad \omega = k_z\,u_A \tag{S3}$$

The corresponding Hamilton equations of motion are

$$\dot{x} = \frac{p_x}{m} - \frac{q}{m}\frac{b_\omega}{k_z}\sin(k\,x - \omega\,t) \tag{S4}$$

$$\dot{p}_x = \left(p_x - q\frac{b_\omega}{k_z}\sin(k\,x - \omega\,t)\right)\frac{b_\omega}{k_z}k\frac{q}{m}\ \cos(k\,x - \omega\,t) - \frac{q^2 B_0^2}{m}x = \dot{x}\frac{b_\omega}{k_z}k\,q\cos(k\,x - \omega\,t) - \frac{q^2 B_0^2}{m}x \tag{S5}$$

As a result,

$$\ddot{x} = \frac{\dot{p}_x}{m} - k\dot{x}\frac{b_\omega}{k_z}\frac{q}{m}\ \cos(k\,x - \omega\,t) + \frac{q}{m}\frac{b_\omega}{k_z}\omega\cos(k\,x - \omega\,t) = -\frac{q^2 B_0^2}{m^2}x + \frac{q}{m}b_\omega u_A\cos(k\,x - \omega\,t) \equiv -\Omega^2 x + \Omega\frac{b_\omega}{B_0}u_A\cos(k\,x - \omega\,t) \tag{S6}$$

Eq. (S6) is the equation of motion resulting from the 1-dimensional Hamiltonian

$$H_1 = \frac{p^2}{2m} + m\Omega^2\frac{x^2}{2} - \frac{b_\omega}{B_0}\frac{m\,\Omega\,u_A}{k}\sin(k\,x - \omega\,t) \tag{S7}$$

Finally, we make expressions (S6, S7) dimensionless through the redefinition

$$x \to k\,x,\;\; t \to t\,\Omega,\;\; p \to p\,\frac{k}{m\,\Omega}\;,\;\; H_1 \to H_1\frac{k^2}{m\,\Omega^2},\;\; \omega \to \frac{\omega}{\Omega} \qquad .$$

Thus

$$\ddot{x} = \; -x + A\,\cos(x - \,\omega\,t) \;\;, \tag{S8}$$

$$H_1 = \frac{p^2}{2} + \frac{x^2}{2} - A\sin(x - \,\omega\,t) \tag{S9}$$

In Eqns. (S8, S9) the normalized wave amplitude *A* is

$$A = \frac{b_\omega}{B_0}\frac{k}{\Omega}u_A = \frac{b_\omega}{B_0}\frac{k\rho}{\Omega\rho}u_A = \frac{b_\omega}{B_0}\;k\rho\frac{u_A}{c_s}\;,$$

as given in the main text by Eq. 1.
