# Supplementary material for: Relevant heating of the quiet solar corona by Alfvén waves: a result of adiabaticity breakdown
Source: Sci Rep. 2019 Oct 3;9:14274. doi: 10.1038/s41598-019-50820-x (PMC6776755; doi:10.1038/s41598-019-50820-x)
Supplement: Supplementary file 4 — Dataset1 [file 41598_2019_50820_MOESM4_ESM.pdf]

Relevant heating of the quiet solar corona by Alfvén waves : a result of adiabaticity breakdown

D.F. Escande,[1] V. Gondret,[2] and F. Sattin[3]
[1] *Aix-Marseille Université, CNRS, PIIM, UMR 7345, Marseille (France)*
[2] *École Normale Superieure, Physics Department, Paris (France)*
[3] *Consorzio RFX (CNR, ENEA, INFN, Università di Padova, Acciaierie Venete SpA), Padova (Italy)*

Supplementary Figures

Figure S1

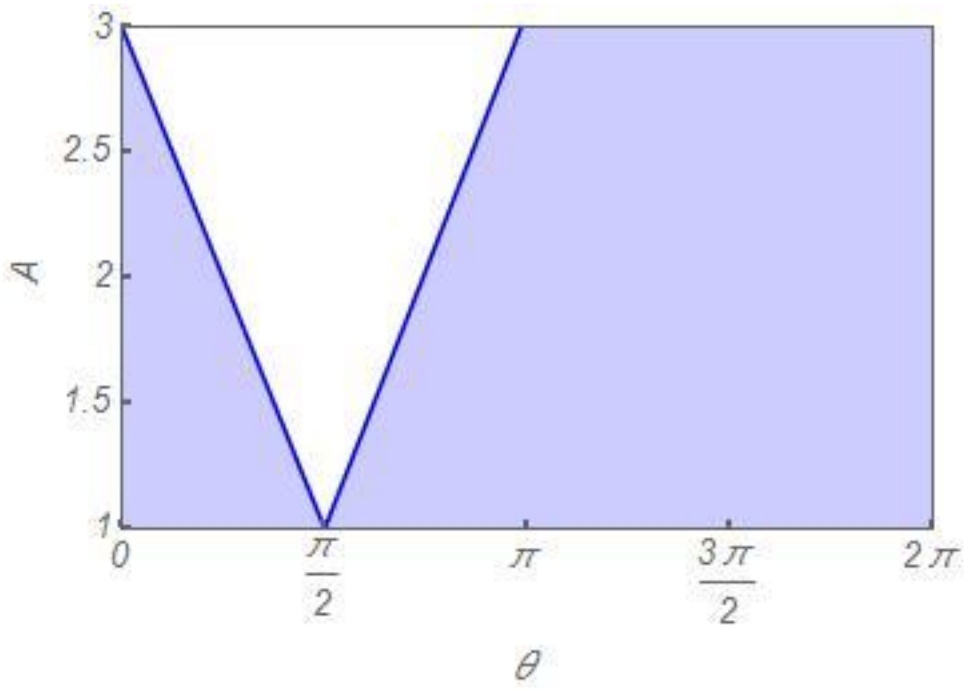

The white region singles out the values of the couple $(\theta = \omega t, A)$ where an X-point exists at $x = X$, as defined by the two conditions $X - A\cos(X - \omega t) = 0$ and $1 + A\sin(X - \omega t) < 0$. The region reduces to the single point $\theta = \pi/2$ when A = 1, and encompasses the whole range (0, 2 π) at $A \approx 5$, when the second X-point appears.

Figure S2

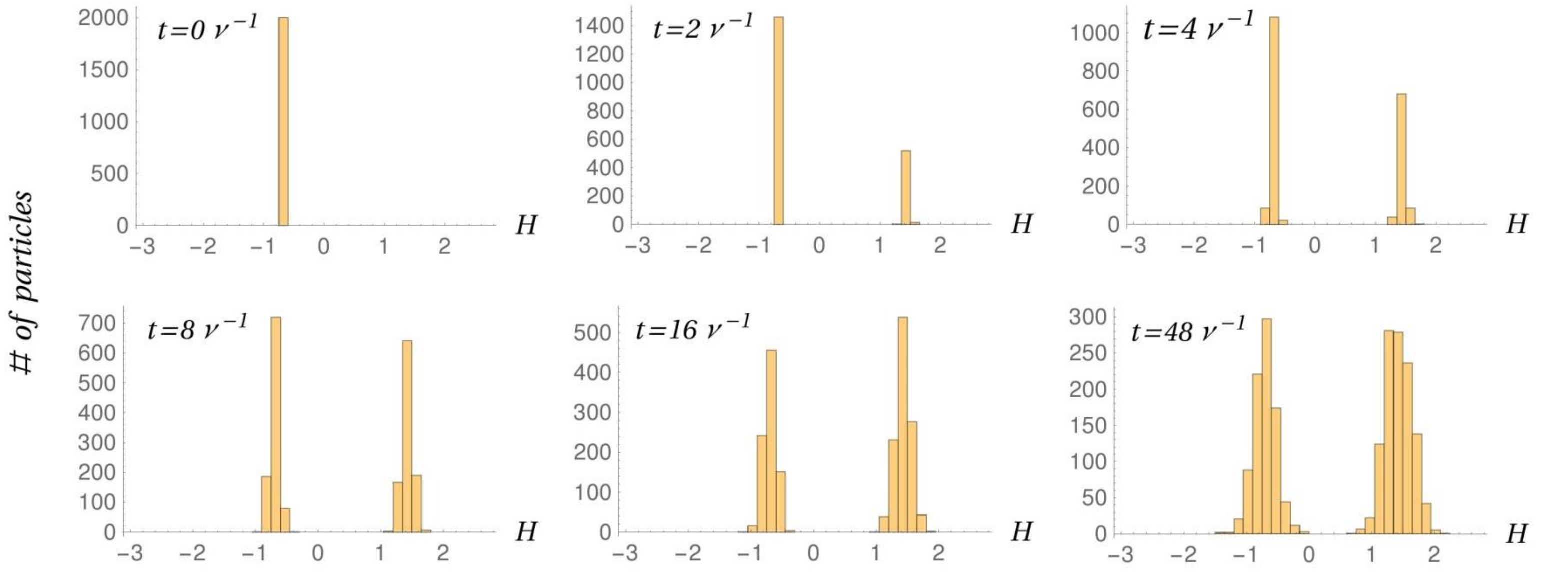

This figure has the same content as Fig. 4 in the main text, but for a different phase of the wave. The particles still distribute into two energy groups; the numerical values are different but the energy gap $E_{high} - E_{low}$ remains of the same order.

Figure S3

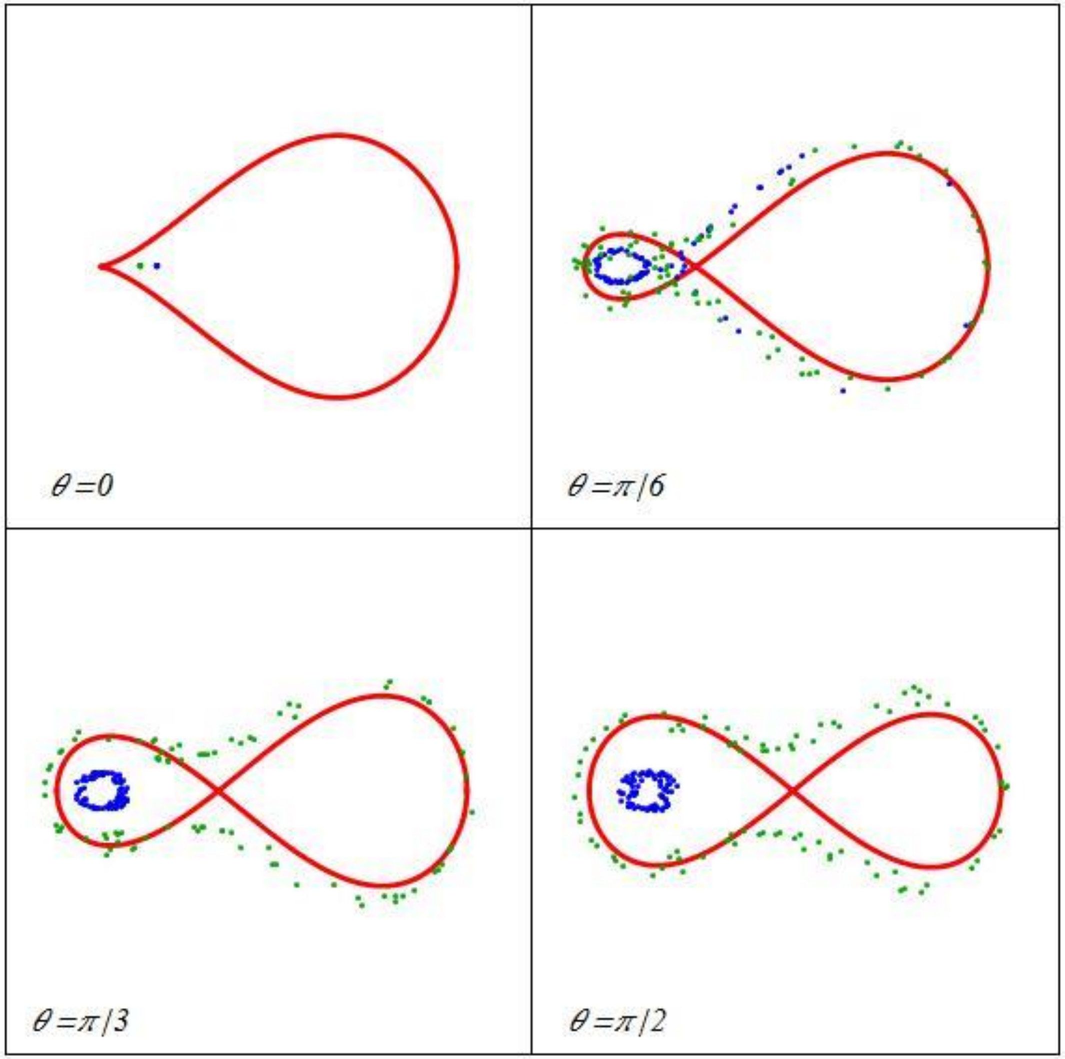

Stroboscopic plots showing the abrupt separation of orbits. Each frame shows the instantaneous position of the slowly pulsating separatrix (the red solid curve) at intervals of $\Delta\theta = \omega\,\Delta t = \pi/6$. Two orbits (green and blue dots) are initialized with marginally different positions near the separatrix in the frame $(\theta = 0)$. The other frames display the stroboscopic plots of the two trajectories, *i.e*. the couples (*x*,*p*) recorded at equispaced times $\Delta t = 2\,\pi$ within the interval $(\theta - \Delta\theta) < \omega\, t < \theta$. Both trajectories cross the separatrix during frame 2; the blue orbit lands into the left lobe and the green one remains stuck to the separatrix when the latter further evolves (frames 3 and 4). The wave parameters are *A* = 3, ω = 0.001, hence each frame contains about 80 points.

Figure S4

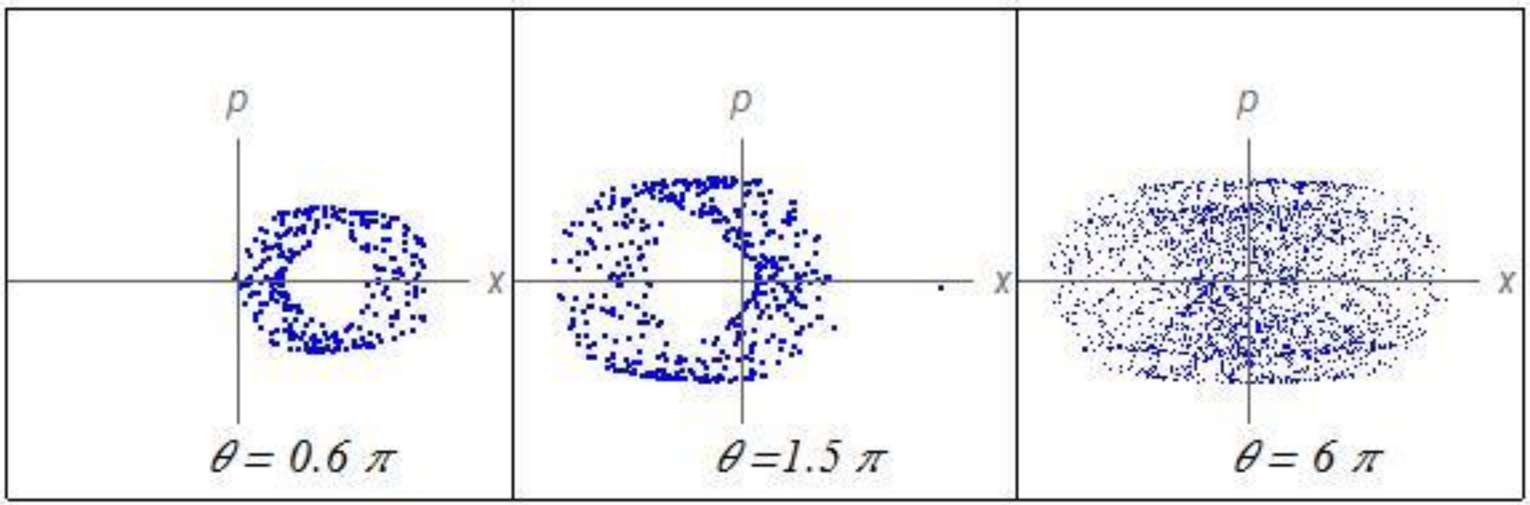

Stroboscopic plots for a single particle trajectory. The wave parameters are those of previous Fig. S3. The origin is the initial position of the particle, at $\theta = 0$. The frames show (*x*,*p*) couples recorded at equispaced times $\Delta t = 2\,\pi$ throughout the ranges $0 < \Delta\theta < 0.6\,\pi$; $0.6\,\pi < \Delta\theta < 1.5\,\pi$; $1.5\,\pi < \Delta\theta < 6\,\pi$ respectively.

Figure S5

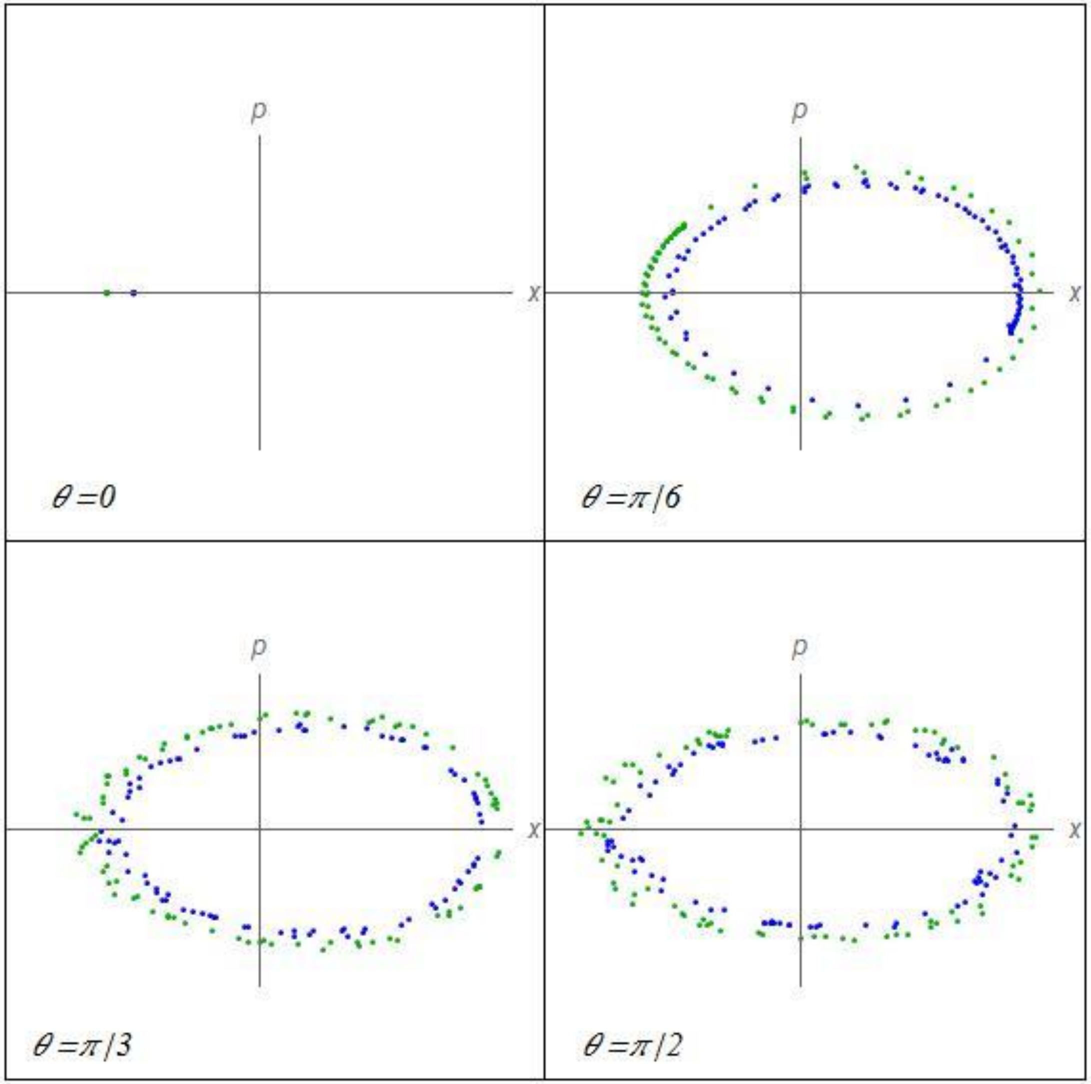

Stroboscopic plots showing the absence of separation of orbits for *A* small. The content of the frames is the same as in Fig. S3. The wave parameters are A = 0.5, ω = 0.001, hence there is no separatrix

Figure S6

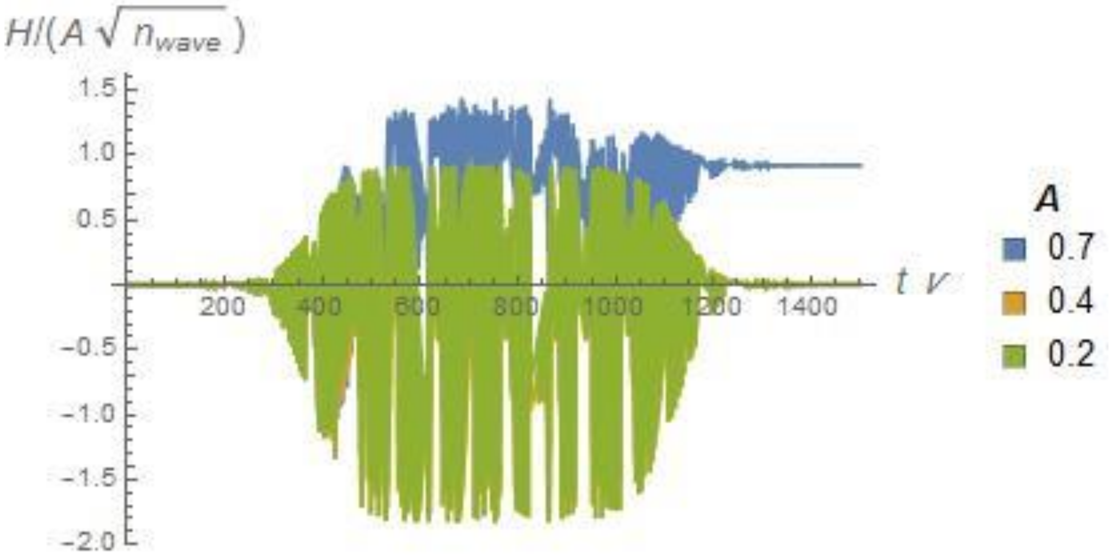

Particle energy gain in a broad spectrum. This figure has the same meaning as Fig. 3 in the main text, but now the spectrum corresponds to $n_{wave} = 5$ waves with the same perpendicular wavenumber, *k* = 1, equispaced frequency $0.01 < \omega < 0.05$, randomly distributed phases, and identical amplitude $a_k \equiv A$, just like done in [C.B. Wang, C.S. Wu and P.H. Yoon, Phys. Rev. Lett. **96**, 125001 (2006)]. The amplitude is turned on and off with a rise time $1500 \times 2\pi$: slow with respect to all the frequencies involved. Just like in the single-wave case, there is irreversible energy transfer when the wave amplitude grows up to values of order unity, but now *A* may be lesser than 1 because of constructive interference effects.

Figure S7

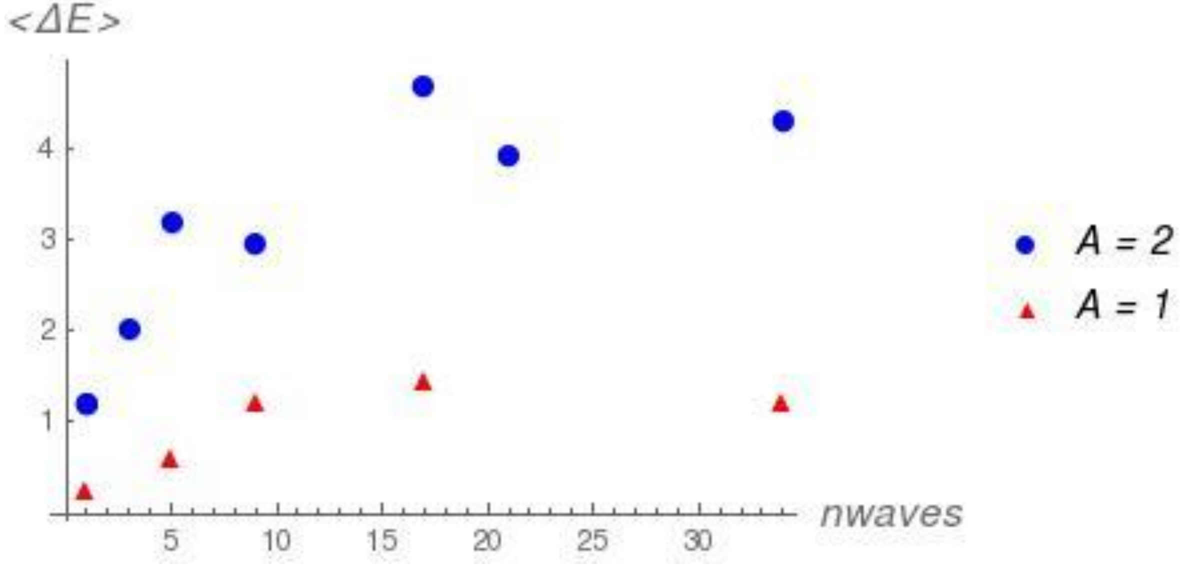

Average energy gain in a broad spectrum. The spectrum is made up of n_{waves} waves, with the same wavenumber, *k* = 1. The frequencies ω are equispaced in the interval (0.01,0.05). The amplitudes are set as $a_k = A/\sqrt{n_{waves}}$ in order to keep energy flux constant between simulations. This spectrum is patterned after [C.B. Wang, C.S. Wu and P.H. Yoon, Phys. Rev. Lett. **96**, 125001 (2006)]. For each simulation 32 independent runs are performed by randomly choosing the phases φ_k, and the energy gain is averaged upon them. There is an increase by roughly a threefold factor when passing from monochromatic (n_{waves} = 1) to broad spectra (n_{waves} > 1), but eventually there is a saturation of the result when n_{waves} >> 1.
